# Developing and Evaluating a Digital Pathology Platform for Veterinary Students: A Case Study in Romania

**DOI:** 10.3390/vetsci12080769

**Published:** 2025-08-17

**Authors:** Bogdan Gabriel Fuerea, Raluca Ioana Rizac, Andrei Robert Botez, Manuella Militaru

**Affiliations:** Paraclinical Sciences Department, Faculty of Veterinary Medicine, University of Agronomic Sciences and Veterinary Medicine of Bucharest, 105 Splaiul Independenței, District 5, 050097 Bucharest, Romania; bogdan.fuerea@edu.usamv.ro (B.G.F.); manuella.militaru@fmvb.usamv.ro (M.M.)

**Keywords:** digital education, e-learning, veterinary learning, whole slide images, digital microscope slide

## Abstract

This paper reports on the development and implementation of a digital pathology platform aimed at improving veterinary students’ learning experiences. Traditional pathology labs limit individual access to physical slides, restricting independent study and detailed examination. The platform provides on-demand access to a comprehensive library of high-resolution digital slides, enabling flexible and personalized learning. Using a mixed-method approach, including surveys and usage analytics, we evaluated its impact on student outcomes. Results showed a significant increase in student engagement and satisfaction, with median usefulness ratings of 5 on a 5-point scale. Usage data indicated enhanced study habits, including increased incidence of students reviewing digital slides independently compared to traditional methods. Qualitative feedback highlighted the platform’s high-resolution imaging and zoom functionality as key features improving students’ understanding of histopathological details. Minor challenges during digitization were addressed to optimize accessibility and interactivity. Overall, this digital platform supports the development of essential diagnostic skills and aligns with contemporary veterinary education skills. The findings demonstrate that integrating digital pathology resources can effectively complement conventional teaching, fostering deeper learning and greater flexibility in study practices.

## Simple Summary

Many veterinary students benefit from innovative teaching tools that enhance their learning experience, especially in complex subjects such as pathology, where visual study materials are essential. Inspired by the potential of digital resources to support and motivate students, we developed an affordable digital platform that provides permanent, 24/7 access to high-quality virtual slides on students’ own devices. This platform was introduced to evaluate how greater flexibility and accessibility influence students’ engagement and understanding. Feedback showed that students valued the opportunity to study at their own pace and found the digital slides helpful in clarifying challenging concepts. They also reported increased motivation. This study highlights how integrating digital tools can enrich veterinary education and support students in building deeper knowledge and skills. Such approaches contribute to advancing the quality and inclusiveness of veterinary training, preparing future veterinarians for success.

## 1. Introduction

The integration of technology into educational environments has evolved dramatically over the last several decades, transforming teaching methodologies and learning experiences across disciplines. As early as the mid-1990s, pioneering efforts to incorporate computer-aided learning in veterinary education were already underway, as exemplified by Holmes and Nicholls’ work [1] at the University of Cambridge, where they developed computer-based learning modules to enhance traditional veterinary education approaches. This early adoption represented the initial phase of a broader technological revolution in educational methodologies, which has since progressed from basic computer-assisted instruction to sophisticated simulations [2,3], virtual laboratories [4], and most recently comprehensive digital platforms that facilitate remote access to educational resources [5]. The trajectory of technological integration in education has been neither linear nor uniform, but rather characterized by periods of rapid innovation interspersed with gradual implementation and adaptation to specific disciplinary needs and pedagogical objectives [6,7,8].

Specific literature can be found on attempts of improving learning materials in our University [9,10] and teaching methods [11,12,13], demonstrating that there will always be a force that works to catch up with current technologies and studies. Furthermore, studies on educational improvements, such as that by [14], emphasize the psychological and social dimensions of technology adoption, investigating student and teacher perceptions of online communication rather than solely focusing on technical implementation.

Veterinary pathology education traditionally relies on microscope-based examination of histological slides, a method that has proven effective for generations of students, but comes with inherent limitations. Among these limitations, perhaps two of the more significant are the physical and time constraint of viewing the slides only during the laboratories. This scenario, common in many veterinary schools worldwide, restricts individual learning experiences and creates an educational bottleneck that can impede comprehensive understanding of pathological concepts. The development of digital pathology platforms represents a transformative approach to addressing these long-standing challenges, offering unprecedented accessibility and educational flexibility to veterinary students [15]. Digital pathology, which involves the use of digital images derived from scanned glass slides, has the potential to revolutionize veterinary education. While the advantages of digital pathology were recognized many years ago, with researchers like Rigaut [16] exploring methods for image analysis and Silage and Gil [17] developing techniques for processing large sections, it is only recently that advancements in slide scanners and software have made widespread implementation feasible. This current resurgence is due to the advancements of slide scanners and viewing software that made the use and implementation of digital pathology feasible in the classroom and research.

This study investigated the hypothesis that digital pathology platforms and digital slides enhance veterinary pathology learning by overcoming traditional limitations. By increasing accessibility and flexibility, digitalization allows for personalized learning, real-time feedback, and collaborative engagement. We aimed to provide evidence on how digital pathology improves educational effectiveness and student engagement in veterinary pathology.

## 2. Materials and Methods

### 2.1. Digital Slide Acquisition

Digital slides were generated using a Grundium™ Ocus^®®^ 40 scanner (Grundium Oy, Tampere, Finland). This scanner was selected based on its scanning speed, cost-effectiveness, portability, and integrated web interface, which allows for the storage and viewing of a substantial number of scanned slides on any device. The scanner settings were optimized to produce high-resolution images suitable for diagnostic and educational purposes, considering the laboratory’s average caseload per year and the selective scanning approach employed.

### 2.2. Storage System Development

Due to the scanner’s limited internal storage and lack of secure sharing capabilities, a custom system was developed for managing and accessing the digital slides. This system comprised the following key components.

#### 2.2.1. Central Storage

A network attached storage (NAS), Synology model DS1621+ (Synology Inc., Taipei, Taiwan)was implemented to serve as the central storage repository for the digital slides. The NAS was initially configured with two 12 TB NAS-specific hard drives in a Synology Hybrid RAID (with data protection for 1-drive fault tolerance) configuration to ensure data redundancy and protection. The NAS model allows for expansion via two expansion units, potentially increasing the total storage capacity to 16 bays.

#### 2.2.2. Database Implementation

An NoSQL database, MongoDB, was chosen for managing case data. This decision was based on the flexibility required to handle various data types and the need for an open-source solution. Data from a previously compiled Excel file (containing 16,983 records from 1997 to 2020) documenting cases were cleaned, standardized, and imported into the MongoDB database. Data validation procedures were implemented to ensure data quality.

#### 2.2.3. API and Web Interface

Python version 3.10 with FastAPI was used to create a robust and efficient API (application programming interface) for interacting with the MongoDB database. Libraries such as NumPy and Pandas were employed for statistical analysis and data manipulation. A front-end web interface was developed using JavaScript and TypeScript with Nuxt3 and TailwindCSS. This combination provided a responsive and user-friendly platform for accessing and managing the digital slides and associated case data.

#### 2.2.4. Web Server and Security

The NAS device’s integrated web server serves the Nuxt3 front end, provides access to the MongoDB database, and exposes the FastAPI endpoint. A container was configured on the NAS to serve these components. The Synology NAS provides HTTPS support and web services, enhancing security and providing a secure domain for access.

#### 2.2.5. Network Configuration

To enable access from outside the local network, specific ports were opened on the router, using port forwarding techniques.

#### 2.2.6. Access and User Management

The Grundium™ Ocus^®®^ scanner is accessible via its web interface (grundium.net) to authorized faculty members only. The NAS-based system is accessible locally within the laboratory network and remotely via a dedicated website. Students and faculty can view slides stored on the NAS. Faculty members have password-protected access to add and modify records in the laboratory database.

**Figure 1:**
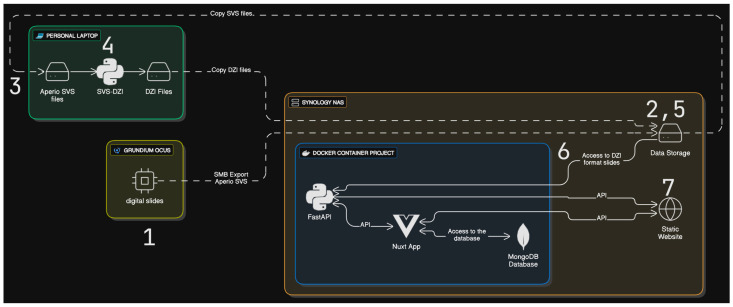
Current software architecture of the digital slides platform.

The system supports the following workflow (Figure 1).

**Figure 2:**
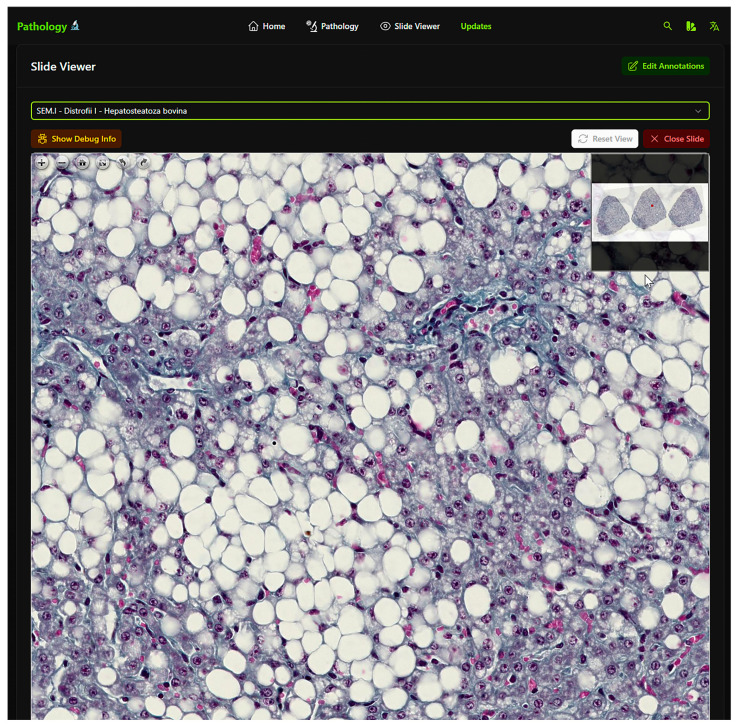
Student platform interface showing a selected slide.

Digital slides were created using the Grundium™ Ocus^®®^ scanner (1), slides being scanned by any of the authorized members. The scanned slides are then exported from the Grundium™ Ocus^®®^ scanner using SMB (server message block) in Aperio slide and viewable storage (SVS) format directly to a specific folder on the Synology NAS for archiving purposes (2). The Aperio SVS files were then retrieved on a high-CPU laptop (3), and converted to deep zoom image (DZI) format using a custom Python script (4). The resulting DZI files were then uploaded to a separate folder on the Synology NAS (5). The Python FastAPI application automatically detected the new DZI files in the designated folder (6) and made them available through the Nuxt3 front end for user access (7). Case data were entered and managed within the MongoDB database. Users accessed the digital slides and associated case data through the web-based interface (Figure 2).

**Figure 3:**
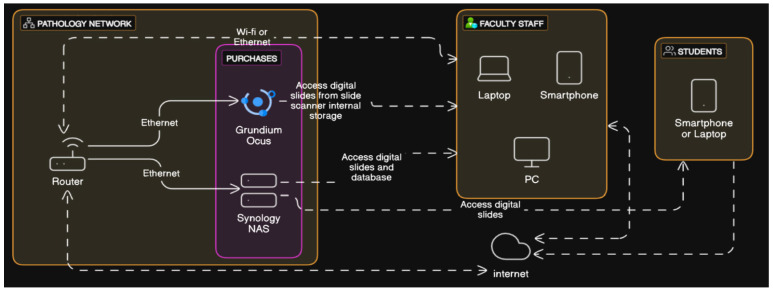
Current hardware architecture of the complete digital lab.

Excepting the initial purchases of hardware, all of the software was either offered alongside the hardware or comprised programming languages and open-source libraries, thus lowering the overall costs. The NAS and the slide scanner are connected to the same network of the pathology laboratory. The faculty staff can access the slide scanner (and its stored digital slides) and the DSP (which is running on the NAS), whereas the students can only access the DSP (Figure 3). Both systems can also be accessed from outside the lab’s network.

One main issue in providing the slides to the students is that the standard open-source programs require more zoom levels for a better viewing. Those additional zoom levels are generated by the python script. The chosen method was the cheapest in term of processing power cost. Converting them once using an additional device frees up the NAS usage and improves loading time for each access, since the files are readily available and the storage space trade-off is still better than an uncompressed SVS file.

### 2.3. Data Preparation

The study was completed using two-phase questionnaires addressed to 3rd year students studying pathology at the Faculty of Veterinary Medicine within the University of Agronomic Sciences and Veterinary Medicine of Bucharest (UASMVB).

Ethics approval for this study was obtained from the UASMVB Ethics Subcommittee, and the document (Conformity Certificate number 4317 CEU29/28.07.2025), both original and translated, can be found in the Supplementary Materials. Both questionnaires secured informed student consent for research purposes, and all participation was anonymous and voluntary. All materials and data utilized in the study adhered to ethical guidelines, ensuring transparency and respect for the rights and interests of all involved parties.

Both questionnaires were distributed to all 242 students. The first questionnaire obtained 192 answers (total *n_I_* = 192), whereas the second obtained 176 answers (total *n_II_* = 176).

The initial phase (I) of our study aimed at collecting baseline data on student perceptions and preferences regarding various educational strategies. By distributing a questionnaire (Table 1) prior to introducing any digital pathology slides as supplementary learning resources, we sought to establish a foundation for analyzing changes in attitudes and preferences after exposure to this approach. The question used, “What methods do you use to prepare for the histopathology test?” (I.1), was designed to evaluate which resources the students consulted. Participants were asked to select one or more methods from a list (Table 2) that included “Learning from the practical workbook” (A), “Reading the notes taken during the laboratory” (B), “Learning with own laboratory sketches” (C), and “Coming to look at slides outside the program” (D). Students then rated the educational utility of each selected method (I.2: I.5) using a five-point Likert scale (1: “Strongly disagree,” 2: “Disagree,” 3: “Neutral,” 4: “Agree,” 5: “Strongly agree”) and finally provided their prospective opinion on the usefulness of digital slides (I.6).

Ahead of Phase II, students were provided with a concise set of instructions to orient them to the DSP. The message highlighted the platform’s intuitive design and covered core functionalities necessary for immediate use, including the access link, the process for selecting specific slides from a dropdown menu, and methods for zooming and viewing slides seamlessly on both personal computers and mobile devices.

In the second phase (II) of our investigation, after the students had access to the digital slides platform (DSP), we administered a follow-up questionnaire (Table 3) to evaluate its impact and gather feedback. This questionnaire validated if the student actually used the platform (II.1), requested a rating for its usefulness (II.2), and then proceeded to obtain positives and improvements ideas based on the students’ direct experiences via two open-ended questions: “What are the positive aspects of the platform? (Why do you think it is good?)” (II.3) and “How do you think the platform could be improved?” (II.4). The next question was focused on the study methods of each student (II.5), establishing in which combination the DSP was used more often, and used options from Table 4. This approach allowed us to assess how the introduction of digital slides affected traditional learning methods and identify opportunities for refining the digital tool. The findings from this phase not only highlighted the effectiveness of integrating technology into educational strategies but also provided valuable insights for future improvements.

### 2.4. Statistical Analysis

Given the nature of the data (Likert-scale usefulness scores and multiple-choice method selections), the following statistical methods were employed.

#### 2.4.1. Descriptive Statistics

Frequencies and percentages describe students’ selections for the multiple-choice study method question. Central tendency is summarized using medians robust to outliers in ordinal data, and variability was measured by interquartile ranges (IQR).

#### 2.4.2. Inferential Statistics

The Kruskal–Wallis test was used to compare usefulness score distributions across the four study methods to identify statistically significant differences. Significant results were followed by post hoc pairwise comparisons using Dunn’s test. Categorical data relationships and distributions were assessed via chi-squared (χ^2^) tests for association and goodness of fit, with p < 0.05 indicating significance. Post hoc analysis employed standardized residuals to pinpoint categories contributing most to significant χ^2^ results, allowing identification of cells with frequencies that deviated substantially from expectations and enabling nuanced interpretation.

### 2.5. Practical Session Structure

Before introducing digital slides, pathology practical sessions followed a conventional format. Each week, teachers presented the topic and students received sets of histopathological slides. The teacher projected slide images with real-time explanations, while students concurrently examined physical slides under microscopes and produced annotated sketches based on the teacher’s instructions. The key steps were: (1) microscopic examination of physical slides; (2) projection and discussion by the teacher; and (3) student sketching and annotation.

Following implementation, the session structure remained unchanged, with digital slides provided as optional supplementary study materials.

## 3. Results

### 3.1. Questionnaire I Results

Analysis of the responses to the first questionnaire’s questions was separated in three stages: I.1, I.—I.6, and a combination of the examination results between the first two.

The provided data reveal interesting insights into how veterinary students prefer to engage with their learning materials and the perceived effectiveness of these methods.

#### 3.1.1. Preferred Study Methods—Question I.1

**Figure 4:**
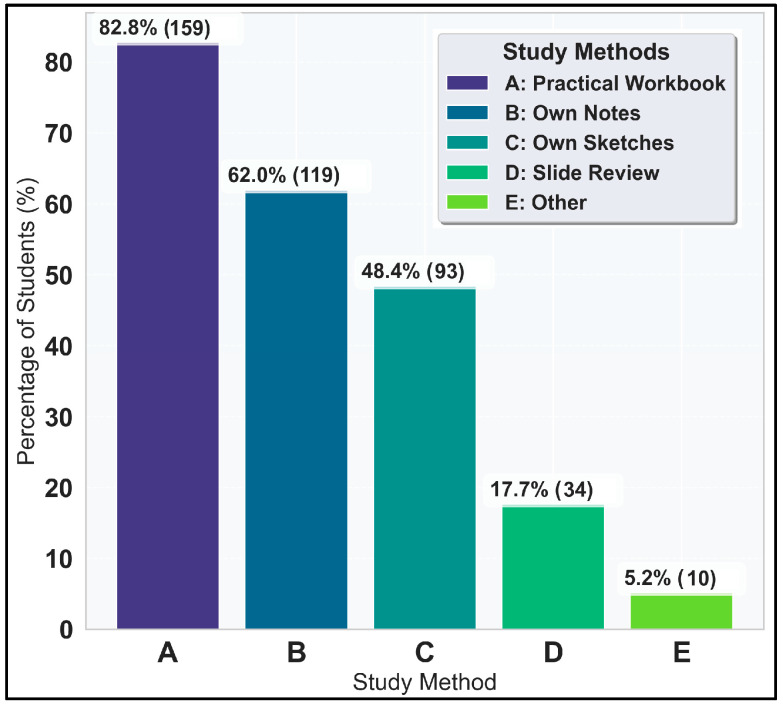
Percentage of occurrence of each study method (individual).

When looking for the occurrences for individual options anywhere in the dataset (Figure 4), practical workbook (A) emerges as the most popular method, employed by 159 students or 82.8% of the respondents. Own notes (B), chosen by 119 participants or 62.0%, are another preferred method. Own sketches (C) are used by 93 students or 48.47% of the participants. Slide review (D) outside class is reported by only 34 respondents or 17.7%.

**Figure 5:**
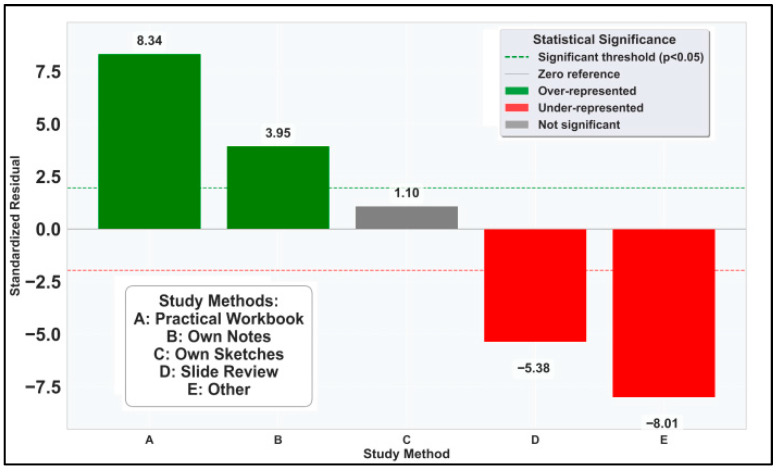
Standardized residuals for individual study methods.

The implementation of the χ^2^ test for individual study methods, with a result of 108.67 (*p* < 0.001), complemented by standardized residuals analysis (Figure 5), indicates that students have a clear preference for certain study methods over others. The magnitude of standardized residuals (>|2.58|) for four methods exceeds the threshold for significance at α = 0.01, confirming robust preference stratification.

Learning from practical workbooks showed extreme over-representation (std. residual = +8.34) whereas the generic “other” methods were extremely under-represented (std. residual = −8.01), suggesting high reliance on available study methods with a focus on using the practical workbooks. Students’ own sketches showed no significant preference bias (std. residual = +1.10), suggesting neutral student reception.

**Figure 6:**
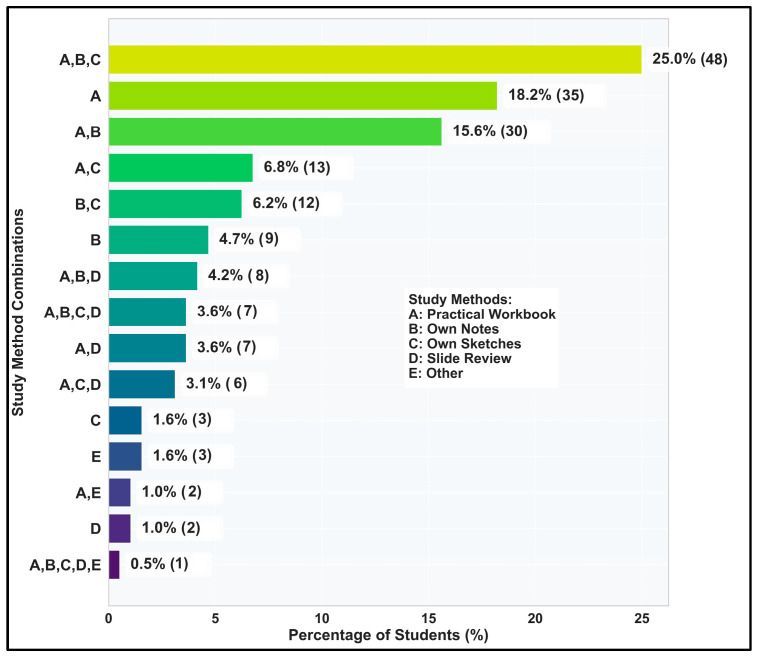
Most common combinations of study methods.

Looking at the most often used combinations (Figure 6), it can be observed that students favored the triad of workbook, own notes, and own lab sketches, indicating a strong preference for organized, textual, and visual learning approaches. Conversely, less structured or comprehensive combinations are significantly less common, suggesting students gravitate towards specific, well-defined learning strategies.

**Figure 7:**
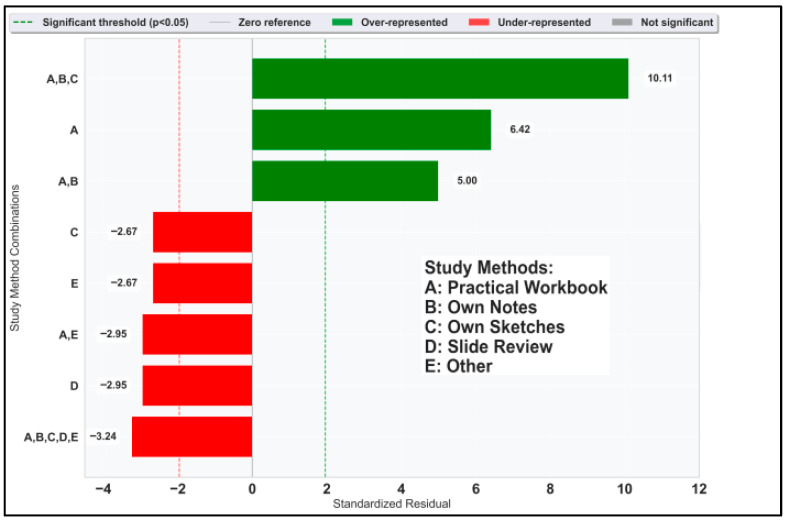
Standardized residuals for notable combinations of study methods.

Undergoing co-occurrence analysis, the χ^2^ value is 71.63 (*p* < 0.001). Individually, students’ own sketches do not stand out in the standardized residual analysis, yet they appear in the most highlighted combination, especially showing extreme overrepresentation (std. residual = 10.11) in the standardized residual analysis for combination of study methods (Figure 7), emphasizing the value of tactile and structured learning modalities in STEM education [8].

#### 3.1.2. Perceived Usefulness of Methods—Questions I.2 to I.5

**Figure 8:**
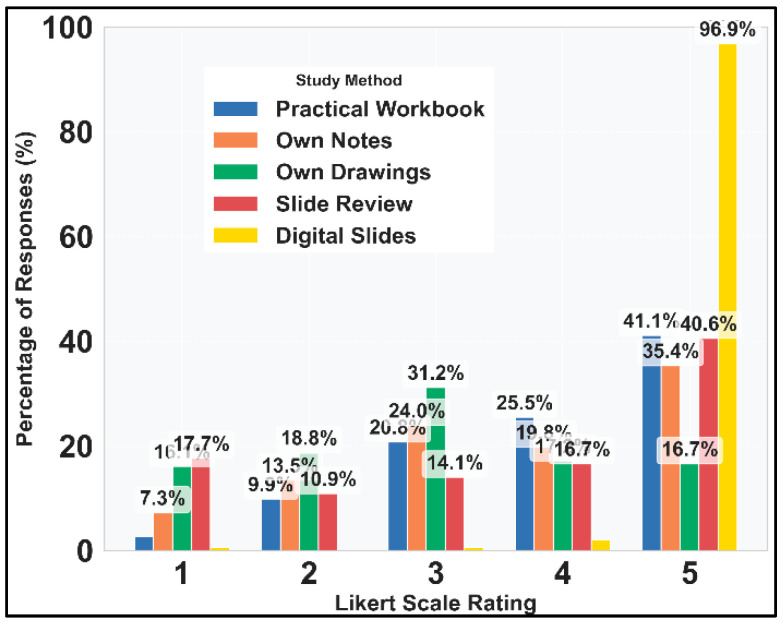
Distribution of Likert-scale responses for study methods.

A first glance at Figure 8 reveals a high expectation for the anticipated use of DSP. The students’ strong anticipation that digital access to histopathology slides would significantly enhance their learning experience represents a clear desire for technological innovation in histopathology education.

**Figure 9:**
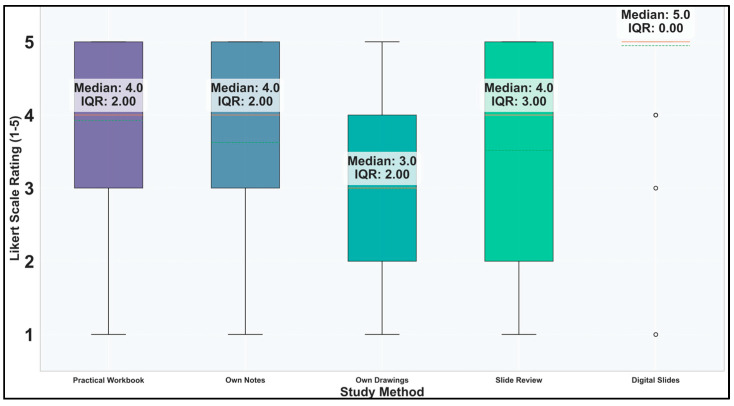
Distribution of responses for study methods. Note: digital slides have prospective scores.

Descriptive statistics (Figure 9) also highlights the enthusiasm for digital slides. Traditional methods like the practical workbook and own notes maintain relatively high ratings (median 4.0), suggesting they still have perceived value. The high IQR for Slide Review (3.0) indicates considerable disagreement among students about its usefulness, with a bimodal distribution (40.6% gave it a 5, while 17.7% gave it a 1). Own sketches received the lowest ratings, which was still above average with a median of 3.

**Figure 10:**
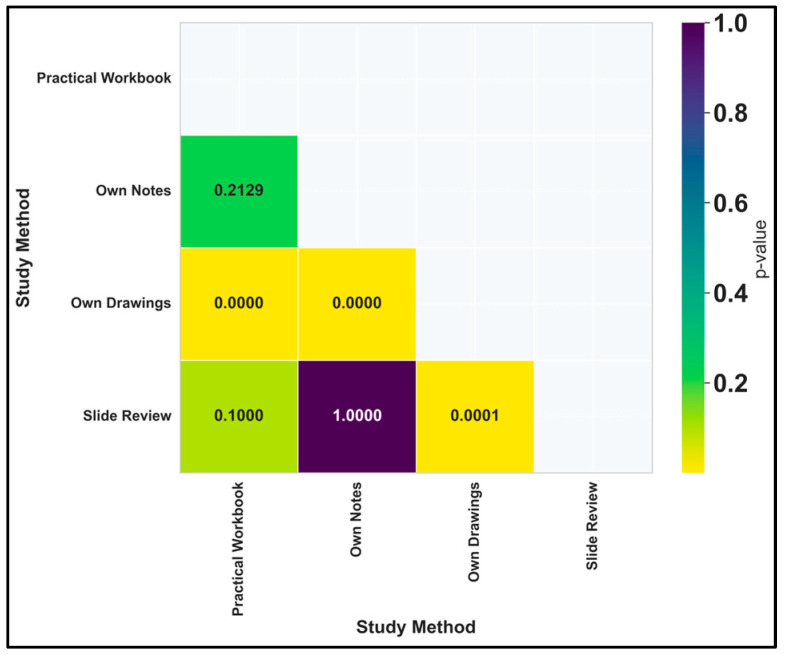
Dunn’s test p-values (Bonferroni-adjusted) for the current study methods.

Excluding the prospective students’ opinion on the DSP and calculating Kruskal–Wallis Test for comparing usefulness, the value of the H statistic was 47.1967 (*p* = 0.0000000003), which reflected that there are statistically significant differences in usefulness ratings across the current study methods. Dunn’s post hoc test for pairwise comparisons (Figure 10) revealed that students have clear preferences among the current study methods. Specifically, own sketches (median 3.0) is rated significantly lower than all other methods (practical workbook, own notes, and slide review, all with median 4.0). The extremely low p-values (*p* ≤ 0.0001) indicate these differences are highly unlikely to have occurred by chance. This means students consistently find drawing histopathology structures less useful than reading the workbook, reviewing their notes, or examining slides directly. The lack of significant differences between the other three methods (practical workbook, own notes, and slide review) suggests students value these approaches similarly. These findings provide strong evidence that drawing-based learning is perceived as less effective than other available study methods for histopathology education.

#### 3.1.3. Comparing the Analysis Results of the First Question with the Likert Scale

One result that poses future questions is the lower rating of own drawings exposed both from question I.1 and I.5, yet high utilization of the combination of the practical workbook and students’ own notes.

### 3.2. Questionnaire II Results

The main question separating the values was to confirm if all students used the DSP (II.1). Only one student reported not accessing the platform.

**Figure 11:**
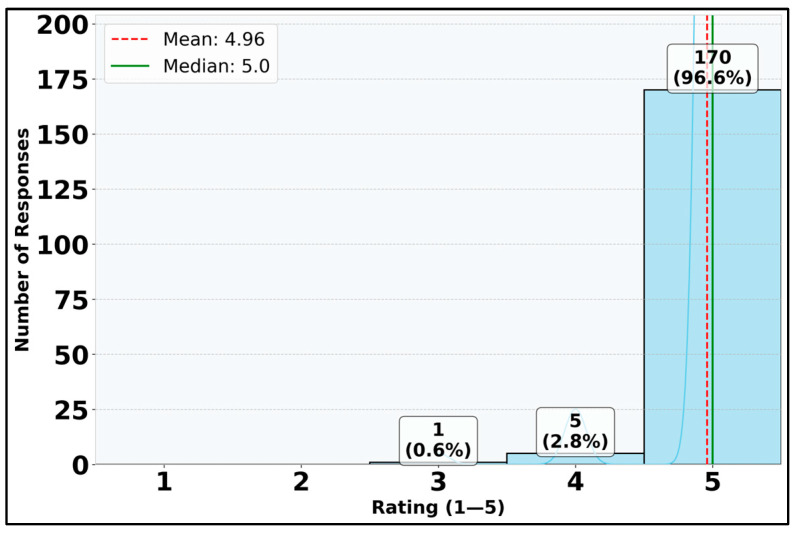
Distribution of responses for DSP usefulness (question II.2).

The analysis of platform usefulness (II.2) reveals exceptionally positive student perceptions. With a median rating of 5.0 and mean of 4.96 on the 5-point Likert scale (Figure 11), students overwhelmingly rated the digital platform at the highest level of usefulness. The distribution is highly skewed toward the maximum rating, with 96.6% of respondents (170 out of 176) giving the platform a rating of 5. The very small interquartile range (IQR = 0.0) and standard deviation (SD = 0.22) indicate remarkable consistency in these positive evaluations. The minimum rating was 3, showing that even the lowest ratings were still positive (Figure 11). These results demonstrate that students found the digital platform to be an extremely valuable educational resource, with near-unanimous agreement about its high utility.

**Figure 12:**
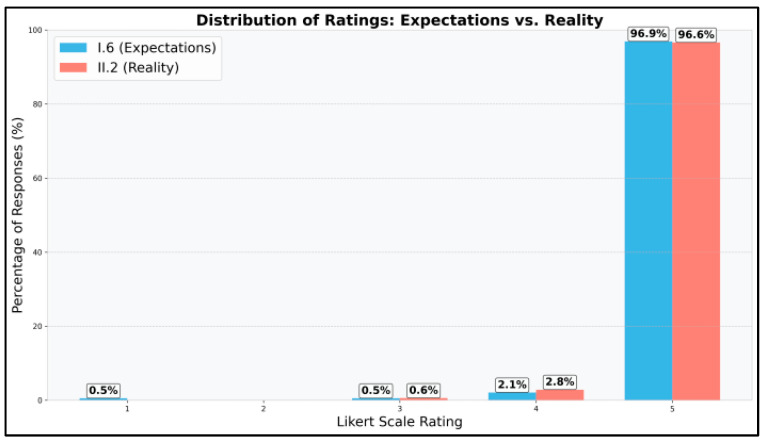
Distribution of ratings: expectations vs. reality for the DSP.

The analysis in Figure 12 compares students’ expectations of the digital platform (I.6) with their actual experience after using it (II.2). The expectations were very high, with a median rating of 5.0 and 96.9% of students giving the maximum rating of 5. After using the platform, the reality matched these expectations, with a median rating of 5.0 and 96.6% of students giving the maximum rating.

The Mann–Whitney U test did not show a statistically significant difference between expectations and reality (*p* = 0.8884). This suggests that students’ actual experience with the platform generally met their high expectations.

For the question probing for the positive aspects of the DSP (II.3), six main categories were manually observed alongside empty and miscellaneous answers:

- Improves the overall learning process.
- Specifically improves the students’ self-review process.
- Specifically improves the reviewing knowledge.
- Accessibility/ease of use.
- Students compare and praise the quality of the digital slides images to their own photos taken from in-lab microscope access.
- Students highlight the modern style of learning and the digital aspect of the platform.
- Students praise the quality of the images and the ability to zoom in.

The high engagement for this question, with only four students not providing an answer, underscores the significant value students place on having access to the DSP.

**Figure 13:**
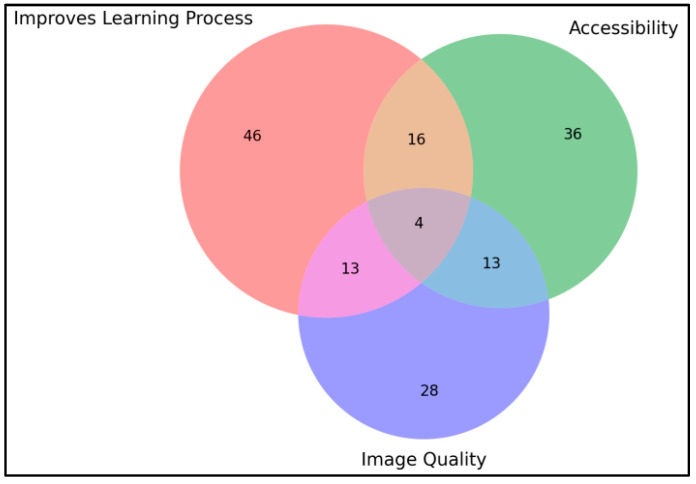
Venn diagram displaying the overlap on response categories for positive-feedback open-ended question (II.3).

The primary categories highlighted by the responses (Figure 13) include the overall improvement of the learning process and accessibility. Students frequently mentioned how the platform enhances their ability to study independently, allowing them to review materials at their own pace and convenience. The accessibility of the platform, enabling students to access slides from anywhere and at any time, was particularly appreciated.

Moreover, the platform’s capability to provide high-quality images and the ability to zoom in on details were repeatedly praised. The digital aspect of the platform, which facilitates a modern approach to learning, was also noted as a significant advantage, bridging the gap between theoretical knowledge and practical application.

**Figure 14:**
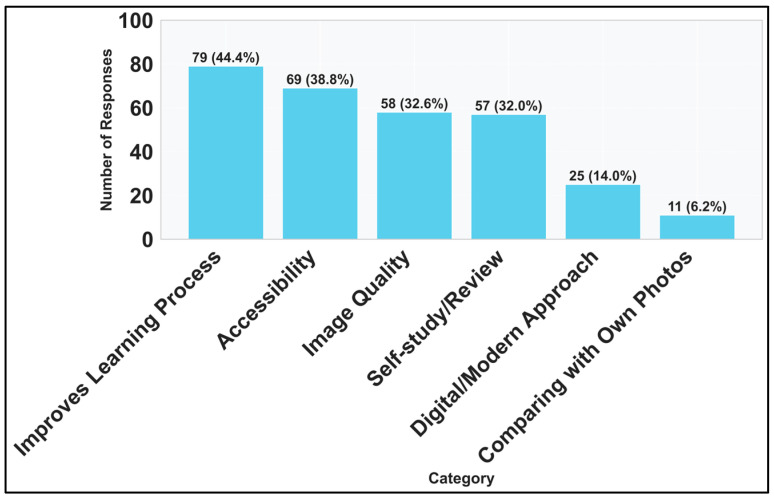
Frequency of categories in responses for positive-feedback open-ended question (II.3).

Table 5 highlights the categories of students’ answers for the positive-feedback open-ended question (II.3), and it is supplemented by Figure 14. Students appreciated the platform’s slide image quality and its accessibility and considered it improved their learning (Figure 14). The connection between the platform’s accessibility, quality and the fact that students compare DSP with their own pictures taken during the laboratory can highlight that they were relying on their own pictures as studying material. If we look at the first phase results, the “Other” section had very low usage. Returning to that data and extracting microscope pictures reveals only two answers specifying microscopy photos, which in turn needs further discussion.

When extracting students’ opinions on possible improvements to the digital slide platform (II.4), only one category was clearly observed alongside empty and miscellaneous answers: the need for annotations (labels and/or additional information about the slide).

In sum, 36.93% of students added annotations or a very similar concept as an improvement, although a notable 41.5% of students felt that no improvement was needed or they did not have a specific idea for one. A substantial proportion still saw value in enhancing the platform with annotations. It is worth mentioning that students were offered the annotations and other supplementary information during the microscopy time of the weekly practical work, and it is a requirement of the students that they write the annotations on each sketch, which may mean that the students would want to have all of their learning materials in one place, instead of using multiple study methods.

**Figure 15:**
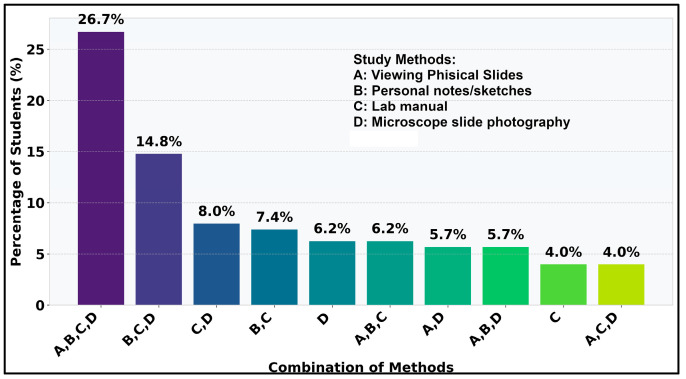
Top 10 combinations of methods used besides DSP.

**Figure 16:**
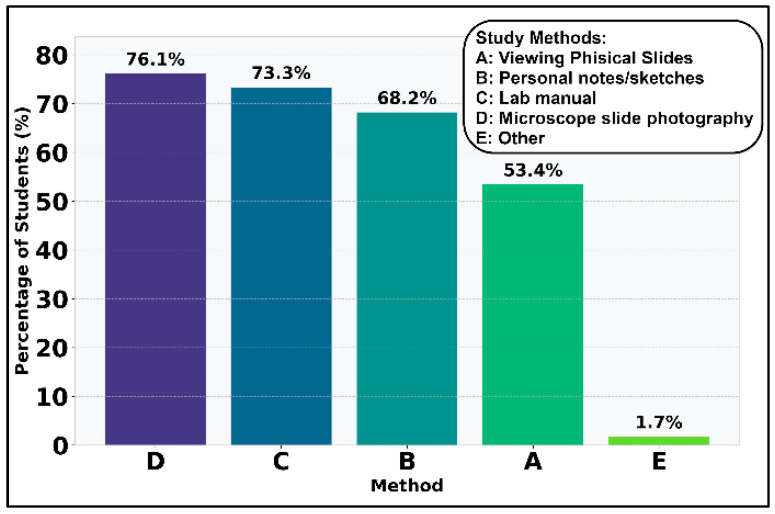
Occurrences of the methods used besides DSP.

The analysis of question II.5 revealed that alongside the DSP, students mostly used all of the available study methods, with 26.7% answers using this combination (Figure 15). Another interesting outcome is the high usage of own microscope pictures (Figure 16), which may show that the students were actually comparing them with the DSP, which actually ties together with the high responses from II.3 about that same topic. This possibility also covers the discussion from the difference in responses mentioning own photos in the initial phase versus the increase of the same in the second phase.

**Figure 17:**
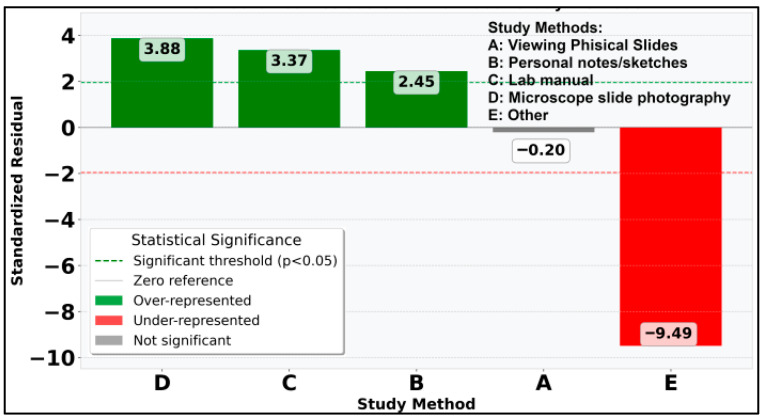
Standardized residuals for individual study methods used besides DSP.

**Figure 18:**
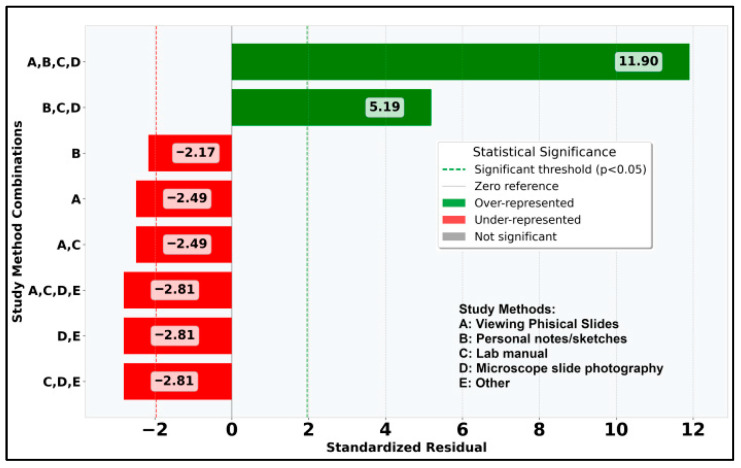
Standardized residuals for notable combined study methods used besides DSP.

Continuing with statistical analysis on the individual and combined study methods used alongside the DSP, it can be observed that χ^2^ value is 101.17 (*p* < 0.0001) so the distribution of individual study methods is statistically significant at α = 0.05. For individual study methods occurrences, the “Other” method is extremely underrepresented with std. residual = −9.49 (Figure 17): only 3 students out of 176 added this option to their answer, which can also be impacted by the use of the DSP. It can also be observed that 26.7% of students (std. residual = 11.90) clearly chose using all available options as additional study materials to the DSP (Figure 18).

## 4. Discussion

### 4.1. Learning Resources and Student Preference

From the first questionnaire it can be established that the preference for the practical workbook likely reflects its structured format, which effectively supports comprehension and navigation of complex histopathological content. This underscores the value of well-organized study materials in enhancing learning outcomes. Note-taking as an active learning strategy emerged as important, reinforcing evidence that learner engagement promotes deeper information processing and retention compared to passive reception. While visual aids such as sketches were rated lower than most, they were still above average and were highly used in combination with the workbook and notes. This is most likely due to the importance of lesions legends which are part of the sketches. Their lower utilization suggests a need to optimize integration of visual learning tools in the curriculum. Limited engagement with slide review outside class indicates logistical barriers, such as restricted access or time constraints, which impede autonomous study. This highlights the necessity of providing flexible, accessible digital resources to facilitate independent learning. Collectively, these insights emphasize that effective educational resources must combine structured content, active learner involvement, and flexible access to address diverse preferences and practical limitations in veterinary education.

### 4.2. Student Expectations and Feedback on the DSP

The extremely high expectations for the DSP suggest that implementing such a platform would be well received, but also creates pressure to deliver a high-quality solution that meets these expectations.

From the second questionnaire, it can be deduced that students appear to have wanted to ensure the DSP can be used with confidence, effectively testing the platform, and once they have confirmed that, some (roughly 35% of the respondents) requested further information to be added to the platform in respect of annotations.

Analysis of the open-ended responses (question II.3) revealed that students consistently rated the DSP favorably, both in anticipation and following its use. These findings suggest that the DSP met or surpassed students’ preexisting expectations.

The flexibility of accessing the slides from any device and from any network not only supported diverse learning styles but also accommodated students’ varying schedules.

Students frequently highlighted the platform’s high-resolution images and zoom functionality, which enable detailed examination of histological structures often overlooked in traditional labs. This enhanced visual access likely contributes to a deeper understanding of the material.

Overall, the feedback indicates that the digital slides platform is an invaluable tool in the students’ educational journey, enhancing both the quality and accessibility of their learning experience. The overwhelmingly positive sentiment expressed in the responses suggests a strong desire among students to continue utilizing this resource as an integral part of their studies.

### 4.3. Implementation and Cost-Effectiveness of Digital Pathology

Our findings demonstrate that digital pathology can be implemented in veterinary education with a focus on cost-effectiveness. By leveraging open-source software and optimizing existing IT infrastructure, institutions can minimize the financial burden of transitioning to digital platforms. This approach aligns with the recommendations of Jones-Hall et al. [7], who suggest that while the initial investment in digital pathology can be substantial—with scanners costing USD 100,000 or more—thoughtful planning and resource allocation can mitigate these costs, making digital pathology more accessible for institutions [6]. To keep costs minimal, IT expertise is required only during the initial setup phase. This underscores the need for an easily deployable open-source solution compatible with standard laboratory hardware—a project currently underway by the author. This project will come with detailed explanations of issues, and overcoming them, similar to the struggles experienced at the stage of scanning older slides, used for teaching purposes due the nature of their important cases, which were thicker than usual, and the scanner was not managing to properly focus and clearly scan them.

### 4.4. Additional Benefits and Applications of Slide Scanners

An additional benefit of acquiring a slide scanner is its ability to enable telepathology, which means our faculty pathologists could access slides from anywhere. This remote access could also be improved by providing it to pathologists in other labs, making it easier to obtain second opinions or work together on cases [6,18]. This functionality has been proven to be unarguably beneficial by Malarkey et al. in 2015 [19], who also highlighted technological limitations at that time. Considering only one student mentioned slowness in accessing the DSP, it suggests that these technical limitations may have largely been overcome.

Another example of projects using slide scanners is the creation of a digital parasite specimen database [20]. This further enhances the benefits of purchasing a slide scanner, as such databases offer advantages like long-term specimen preservation and wide accessibility for educational and research purposes.

### 4.5. Context of Digital Technology in Romania

Digital technology has been researched in various fields in Romania. Notable work has been conducted in digitizing agricultural tools and procedures (see, for example, [21,22,23], with applications ranging from waste classification using neural networks and web applications to machine learning approaches for land use change modeling, and digital systems for monitoring crop canopies. The development of digital platforms to support agriculture, forestry, and food industries is also evident [24]. In the field of biotechnology, Romanian researchers have explored mobile applications and e-learning tools to enhance education and practical outcomes [25,26], as well as the use of cloud computing and digital imaging for animal science and crop analysis [27,28].

Notably, this study appears to be the first in Romania to explore the integration of digital slide technology for veterinary pathology education, underscoring the potential for future research and development in this area within the country. In Romania, a project on human pathology education was discovered, called “Digital transformation of Histology and Histopathology by Virtual Microscopy (VM) for an innovative medical school curriculum,” the VM3.0 project, started between the University of Medicine and Pharmacy, Iasi, Romania and universities from Poland, Bulgaria, Spain, and Greece, co-funded by the European Union [29]. Alongside the Digital Pathology Association [30], they highlight the differences in investments between human and veterinary medicine, thus highlighting the importance of this article’s topic.

### 4.6. Limitations and Future Prospects

One limitation of this study is that the digital platform was implemented exclusively within the pathology discipline and did not extend to other subjects that rely on microscopic slide analysis, such as cytopathological diagnosis. This restricts the generalizability of the findings across disciplines; however, the platform holds potential for future adaptation to other areas of study. Additionally, the development and maintenance of such a platform require a background in information technology. This presents a barrier for educators or institutions lacking the necessary technical expertise, potentially limiting scalability and long-term sustainability without interdisciplinary collaboration.

## 5. Conclusions

The integration of the digital slide platform significantly enhanced veterinary students’ learning experiences and study outcomes. By providing on-demand access to a comprehensive library of high-resolution digital pathology slides, the platform overcomes logistical barriers and supports flexible, self-directed learning. This centralized digital resource facilitates the acquisition of essential diagnostic and interpretive skills through remote examination of histopathological specimens, directly contributing to the achievement of day one skills. These skills are fundamental for graduates entering modern veterinary practice, ensuring proficiency with digital tools integral to the field.

Student feedback has identified priorities for further improvement, particularly regarding interactivity and accessibility, guiding future platform enhancements. Overall, this study demonstrates the substantial educational value of digital slide technology and its pivotal role in preparing students for the practical demands of contemporary veterinary medicine.

## Figures and Tables

**Table 1:** Initial-phase questionnaire.

Identifier	Question (EN)	Type
I.1	What methods do you use to prepare for the histopathology test?	Multiple Choice
I.2	To what extent is the practical workbook useful and do you use it to prepare for the histopathology test?	Likert Scale
I.3	To what extent are your own notes useful and do you use them to prepare for the histopathology test?	Likert Scale
I.4	To what extent are your own sketches useful and do you use them to prepare for the histopathology test?	Likert Scale
I.5	To what extent is it useful and do you use off-schedule slide review under the microscope in the lab for histopathology test preparation?	Likert Scale
I.6	How useful would it be to have scanned and digitally available histopathology slides?	Likert Scale

**Table 2:** Initial-phase multiple choice question (I.1)—Available Options.

Identifier	Option (EN)	Option—Short
A	Learning from the practical workbook	Practical Workbook
B	Reading the notes taken during the laboratory	Own Notes
C	Learning with own laboratory sketches	Own Sketches
D	Coming to look at slides outside the program	Slide Review
E	Other (free-text response)	Other

**Table 3:** Second-phase questionnaire.

Identifier	Question (EN)	Type
II.1	Have you accessed the platform for viewing digital slides?	Dichotomous
II.2	How useful do you think the platform was, on a scale of 1 to 5?	Likert Scale
II.3	What are the positive aspects of the platform? (Why do you think it is good?)	Open-Ended
II.4	How do you think the platform could be improved?	Open-Ended
II.5	Besides the digital platform, what other methods did you use to prepare for the practical histopathology test? (Select all applicable options)	Multiple Choice

**Table 4:** Second-phase multiple choice question (II.5)—available options.

Identifier	Option (EN)	Option—Short
A	Viewing physical slides in the laboratory	Viewing Slides
B	Personal notes/sketches	Own Notes
C	Lab manual	Lab Manual
D	Microscope slide photography	Slide Photo
E	Other (free-text response)	Other

**Table 5:** Interpretation of students’ answers to the positive-feedback open-ended question (II.3). Answers could fit into multiple categories.

Category	Count	Representative Concepts
Generally, improves the learning process	79	Helps or improves study
It’s easy to use/best accessibility/more time to study	69	Easy/easier to learn, accessible, time, efficiency
Praising the zoom capability/Image quality	58	Clear pictures, zoom, picture quality
Highlighting the improvement on self-study/helps reviewing knowledge	57	Specifically mentioning self-study and/or reviewing knowledge
Highlighting the digital aspect and its benefits/It’s a modern study method	25	Modern, digital
Comparing digital slides with own microscope photos	11	Microscope photos, comparison, digital slides
No answer	4	Filler characters and/or spaces only

## Data Availability

The dataset supporting the conclusions of this article is provided in the Supplementary Materials. The custom code used for data analysis and platform development contains sensitive information and is not publicly available at this time; however, the main author is willing to provide additional information or code snippets upon reasonable request. Details on the software and hardware architecture are described within the manuscript.

## Supplementary Materials

The following supporting information can be downloaded at https://www.mdpi.com/article/10.3390/vetsci12080769/s1. From file “Feedback Responses.xlsx”: Table S1: First questionnaire answers; Table S2: Second questionnaire answers; Table S3: Analysis of students’ responses to open-ended question II.3; Table S4: Analysis of students’ responses to open-ended question II.4; Table S5a: Translations for all questions from both questionnaires; Table S5b: Translations for all options from both questionnaires’ multiple-choice questions. From folder “vetsci-12-00769-ethics-proof”: Scientific Research Ethics Subcommittee of UASVM of Bucharest Approval documents—original and translated.
